# Influence of Dietary Undegraded Crude Protein on Intake, Nitrogen Partitioning, and Apparent Organic Matter Digestibility in Alpacas

**DOI:** 10.1111/asj.70176

**Published:** 2026-04-10

**Authors:** Jefone Caleb Alvarez Martinez, Carla Sofía Zaga Alarcón, Jose Luis Contreras Paco, Juan Elmer Moscoso‐Muñoz, Juan Olazabal‐Loaiza, Giovanna Janet Gómez‐Oquendo, Khaterine Cinthia Salazar‐Cubillas

**Affiliations:** ^1^ Faculty of Veterinary Medicine and Zootechnics Universidad Científica del Sur Lima Peru; ^2^ Faculty of Agricultural Sciences National University of Huancavelica Huancavelica Peru; ^3^ Faculty of Agricultural Sciences National University of San Antonio Abad of Cusco Cusco Peru; ^4^ Faculty of Veterinary Medicine National University of San Marcos Lima Peru; ^5^ Institute of Animal Nutrition and Physiology Christian‐Albrechts‐Universität Zu Kiel Kiel Germany

**Keywords:** protein degradability, protein nutrition, South American camelids

## Abstract

The present study evaluated the influence of three dietary levels of undegraded crude protein in the fermentative compartment (UDCP) on intake, fecal and urinary excretion, nitrogen partitioning, and apparent organic matter digestibility in alpacas. Eight male alpacas were assigned to low (14% crude protein), medium (24% crude protein), and high (34% crude protein) UDCP treatments in an incomplete and unbalanced 3‐period crossover design using isoproteic and isoenergetic diets. No differences were observed among treatments in dry matter intake, organic matter intake, fecal excretion of dry matter, organic matter, and nitrogen; nitrogen retention; or apparent organic matter digestibility. In contrast, water intake, nitrogen intake, urinary nitrogen excretion, nitrogen absorbed, and nitrogen efficiency ratios were influenced by UDCP treatment. The high UDCP treatment was characterized by lower urinary nitrogen losses and a higher proportion of retained nitrogen relative to absorbed nitrogen compared with the low UDCP treatment, despite similar nitrogen intake. Conversely, the low UDCP treatment promoted higher nitrogen absorption but was associated with greater urinary nitrogen excretion and a lower retention‐to‐absorption ratio. Overall, these results indicate that increasing the dietary contribution of UDCP reduces post‐absorptive nitrogen losses in alpacas, thereby improving nitrogen utilization efficiency relative to low UDCP diets.

## 1 Introduction

Alpaca farming plays a key role in high Andean production systems, with Peru housing approximately 87% of the global population and leading global fiber and meat production (MIDAGRI 2020). This activity provides a significant source of income for rural communities, where up to 50% of household revenue derives from fiber sales, supplemented by meat and breeding stock (Carpio Valencia 2017). However, diets in these regions rely primarily on low‐quality forages with low crude protein (CP) concentrations (from 2.47% to 5.21%; Quispe Ramos et al. 2021) and a high proportion of degradable CP in the fermentative compartment (from 45% to 88%; data not shown in the present study; Contreras‐Paco et al. 2021), resulting in a limited supply of undegraded CP in the fermentative compartment (UDCP).

Balancing proportions of degradable and UDCP in the fermentative compartment is essential because an excess degradable CP increases ammonia production in the fermentative compartment, leading to greater conversion to urea and urinary excretion, ultimately reducing nitrogen retention (Hristov et al. 2019). While these relationships are well established in true ruminants, where adequate balancing of degradable and UDCP with fermentable energy improves nitrogen utilization and microbial protein synthesis (NRC 2001), evidence in South American camelids remains limited. Only a few studies have evaluated the effects of dietary UDCP proportions on nitrogen partitioning in alpacas (Lund et al. 2012), which restricts the development of species‐specific nutritional recommendations. Therefore, the present study was designed to evaluate the influence of three dietary levels of UDCP on intake, fecal and urinary excretion, nitrogen partitioning, and apparent organic matter (OM) digestibility in alpacas.

## 2 Materials and Methods

All animal procedures were reviewed and approved by the Institutional Committee of Ethics in Research with Animals and Biodiversity of Universidad Científica del Sur (CIEI‐AB‐CIENTÍFICA), Lima, Peru. The study was registered under code PRE 16‐2023‐00232 and approved under certificate number 096‐CIEI‐AB‐CIENTÍFICA‐2023.

The present study was conducted in two stages: pre‐experimental (in situ) and experimental (in vivo). The pre‐experimental stage was carried out in the Laboratory of Animal Science and Agricultural Production of the National University of San Marcos (Lima, Peru). The experimental stage was conducted at the La Raya Experimental Center of the San Antonio Abad University of Cusco (Cusco, Peru). Sample analyses were performed at the Laboratory of Animal Nutrition and Physiology of Kiel University (Kiel, Germany) and at the Laboratory of Animal Nutrition and Feed Evaluation of the National University of Huancavelica (Huancavelica, Peru).

### 2.1 Pre‐Experimental Stage

The pre‐experimental stage aimed to determine the in situ CP degradability of eight feedstuffs in the fermentative compartment of alpacas to formulate diets with known CP degradability. The feedstuffs evaluated were alfalfa hay (*Medicago sativa)*, oat hay ( *Avena sativa* ), forage barley ( *Hordeum vulgare* ), *Festuca dolichophylla*, *Calamagrostis vicunarum* , *Lolium perenne* , soybean meal, and fish meal. Two adult male Huacaya alpacas (54 ± 5 kg body weight), aged 4 and 5 years, were used to conduct the in situ incubations. The alpacas, surgically fitted with fistulas in the first compartment, were housed together in a 40 m^2^ pen. Their diet consisted of a mixture of oat hulls (50%), alfalfa hay (40%), wheat bran (10%), and minerals, with ad libitum access to oat hay and water. All procedures involving animals were conducted in accordance with the Animal Welfare Legislation (PRE‐19‐2023‐00232).

Fresh feed samples were dried at 60°C and ground to particle sizes of 2 mm for in situ analysis and 1 mm for subsequent laboratory analyses. The in situ dry matter (DM) degradability, OM, and CP were measured by incubating the ground feed samples in polyester bags using a protocol adapted from Madsen and Hvelplund (1994). Polyester bags (R1020, Ankom Technology, NY, USA) with a pore size of 53 ± 10 μm were used. Before use, bags were pre‐dried at 60°C for 48 h in a forced‐air oven (LBX Instruments, Model OVF), weighed (gram FS, plate size 9 cm, capacity 120 g, precision ±0.1 mg), and labeled. Each bag was filled with 3 g of dried, 2 mm‐ground feed sample to achieve a sample density of approximately 15 mg/cm^2^, then sealed with plastic cable ties and attached to a 35 cm nylon string.

Prior to incubation, bags were soaked in warm water (~39°C) for 5 min. Incubation times were standardized at 0, 6, 12, 24, and 48 h, except for soybean meal, which excluded the 48‐h point. Replications per time point and alpaca were defined according to minimum sample size requirements based on degradation kinetics in ruminants. Up to 10 bags were introduced into the first compartment of each alpaca at 07:00 h before feeding. After incubation, bags were removed simultaneously, immersed in cold water to stop microbial activity, washed by hand until rinse water was clear, oven‐dried at 60°C for at least 2 h, and weighed to determine DM disappearance. Residual substrate after incubation was pooled by alpaca and incubation time for analysis of crude ash (AOAC 942.05) and CP (AOAC 990.03).

The CP disappearance at different incubation times was fitted to the nonlinear model of Ørskov and McDonald (1979) to describe degradation kinetics for each feedstuff in the fermentative compartment. CP was partitioned into a rapidly soluble fraction (a), a potentially degradable fraction (b), and a fractional degradation rate of the potentially degradable fraction (c). Effective CP degradability was estimated using assumed digesta passage rates of 2%, 5%, and 8% per hour, representing low, intermediate, and high outflow conditions, respectively, from the fermentative compartment.

Based on the results of these analyses, four feedstuffs were selected to formulate the experimental treatments (Tables 1 and 2): alfalfa hay ( *M. sativa* ), harvested at 35 days of growth and collected from Arequipa; oat hay ( *A. sativa* ), harvested at 3 months and collected from Cusco; and soybean meal and fish meal, both sourced from Lima, Peru. These feedstuffs were selected due to their contrasting CP degradability values, which allowed the formulation of experimental treatments with clearly differentiated levels of protein degradability.

**TABLE 1:** Protein degradability of feed ingredients at different passage rates in the fermentative compartment of alpacas.

Feedstuffs	(%)^a^	(%)^b^	(%/h)^c^	EDCP (%CP) 2%/h	EDCP (%CP) 5%/h	EDCP (%CP) 8%/h
Oat hay	39.54	50.34	0.04	73.19	62.09	56.52
Soybean meal	10.40	89.60	0.13	91.11	77.91	68.43
Alfalfa hay	42.72	48.85	0.25	87.45	82.55	78.69
Fish meal	23.28	33.83	0.07	47.94	41.16	37.41

*Note:* The degradation parameters a, b, and c were estimated separately for each alpaca using nonlinear regression, and reported values represent the mean across alpacas; effective degradability at passage rates of 2%, 5%, and 8%/h was calculated per alpaca and presented as mean values.

Abbreviations: CP, crude protein; EDCP, effective degradability of crude protein.

^a^ Rapidly soluble fraction.

^b^ Potentially degraded fraction.

^c^ Degradation rate.

**TABLE 2:** Chemical and nutritional characteristics of individual ingredients.

Ingredients	Oat hay	Soybean meal	Alfalfa hay	Fish meal
*Chemical composition (g/kg DM)*				
Organic matter	985.61	922.45	888.80	875.52
Crude protein	37.15	586.74	225.41	479.65
Neutral detergent fiber	616.70	105.00	364.68	0.00
*Nutritional characteristics (MJ/kg DM)*				
Metabolizable energy	7.00	13.40	9.30	11.50

*Note:* Neutral detergent fiber values for alfalfa hay and fish meal and metabolizable energy values for all feed ingredients were obtained from Feedipedia (INRAE‐CIRAD‐AFZ‐FAO). Abbreviation: DM, dry matter.

### 2.2 Experimental Stage

The experimental stage aimed to evaluate the effect of dietary UDCP on intake, fecal and urinary excretion, nitrogen partitioning, and apparent OM digestibility in alpacas. Three isoproteic and isoenergetic diets were formulated based on in situ degradability results from the pre‐experimental stage, using alfalfa hay, oat hay, soybean meal, and fish meal (Table 3). The coefficients of variation for CP and energy concentration among the three diets were 4.3% and 1.1%, respectively, both below the 5% threshold used to assess reproducibility between replicates. Diets were classified according to their UDCP proportion at a 2%/h passage rate, expressed as a percentage of CP: 14% (low UDCP), 24% (medium UDCP), and 34% (high UDCP) for the present study. The calculations were performed using a passage rate of 2%/h to reflect the prolonged solid mean residence time reported for alpacas in the fermentative compartment, which has been shown to exceed 50 h under forage‐based diets (Yao et al. 2015).

**TABLE 3:** Ingredient inclusion and calculated chemical and nutritional characteristics of experimental diets.

Treatments	Low UDCP	Medium UDCP	High UDCP
*Ingredients (% of inclusion in DM)*			
Oat hay	81.70	86.60	85.70
Soybean meal	11.50	8.40	4.30
Alfalfa hay	6.80	—	—
Fish meal	—	5.00	10.00
*Chemical composition (g/kg DM)*			
Organic matter	971.76	974.80	971.89
Neutral detergent fiber	540.72	542.88	533.03
Crude protein	113.15	105.44	105.03
*Nutritional characteristics*			
UDCP in the fermentative compartment (%CP)	14	24	34
Metabolizable energy (MJ/kg DM)	7.89	7.76	7.73

*Note:* Diet chemical composition was calculated based on the additive contribution of individually analyzed feed ingredients. Neutral detergent fiber values for alfalfa hay and fish meal were obtained from Feedipedia (INRAE‐CIRAD‐AFZ‐FAO). Metabolizable energy values for ruminants for all feed ingredients were obtained from Feedipedia (INRAE‐CIRAD‐AFZ‐FAO).

Abbreviations: DM, dry matter; UDCP, undegraded crude protein in the fermentative compartment of the alpaca.

The experimental stage involved eight male alpacas (2 years old; body weight 52 ± 2.92 kg), which were dewormed with ivermectin (0.2 mg/kg body weight) before the trial. This stage was divided into two phases: adaptation phase and data collection phase. During the 8‐day adaptation phase, alpacas were gradually introduced to metabolic cages equipped with individual feeders and drinkers and were familiarized with daily handling and the experimental feedstuffs to ensure proper adaptation. The data collection phase consisted of three 18‐day periods and was conducted using an incomplete and unbalanced 3‐period crossover design with three treatments (Table 4).

**TABLE 4:** Treatment allocation by period.

Period	Alpaca identification number							
	1	2	3	4	5	6	7	8
1	Low UDCP	Low UDCP	Low UDCP	High UDCP	High UDCP	High UDCP	Medium UDCP	Medium UDCP
2	Medium UDCP	Medium UDCP	Medium UDCP	Low UDCP	Low UDCP	Low UDCP	High UDCP	High UDCP
3	High UDCP	High UDCP	High UDCP	Medium UDCP	Medium UDCP	Medium UDCP	Low UDCP	Low UDCP

Abbreviation: UDCP, undegraded crude protein in the fermentative compartment of the alpaca.

#### 2.2.1 Feed and Water Consumption

Animals were placed individually in 1.60 m long × 0.75 m wide × 2.0 m high metabolic cages. The amount of feed offered was calculated during the adaptation phase by determining the voluntary intake of each animal with feed offered ad libitum (ranging from 2.1% to 2.6% body weight). In the data collection phase, this intake was increased by 10% to ensure refusals. Feeding was conducted at 07:00 h, and feed refusals were collected the following day at 06:00 h prior to the next feeding. The same methodology was applied to measure water intake, with water provided ad libitum.

A total of 1.5 kg of the diet offered, as well as samples of individual ingredients, were collected daily during the data collection phase. Samples were dried at 60°C to constant weight, ground to 1 mm, and analyzed for DM (AOAC 934.01), crude ash (AOAC 942.05), and CP (AOAC 990.03). Voluntary DM intake was calculated as the difference between dried feed offered and dried refusals (López et al. 1998). The concentrations of crude ash and CP in the analyzed samples were multiplied by feed intake to calculate OM intake and nitrogen intake.

#### 2.2.2 Fecal and Urine Sample Collection

Total feces were collected daily during the data collection phase using polyethylene harnesses attached to the animals. Each day, total feces were weighed, and a 200 g subsample was taken, labeled, and stored at −20°C in a freezer (INDURAMA 420 L, CI‐420BL) for later analysis of DM (method 3.1), crude ash (method 8.1), and CP (method 4.1.2) (VDLUFA 2012). The OM apparent digestibility was calculated as the difference between OM consumed and OM excreted, divided by OM consumed.

Total urine was collected daily during the data collection phase using metabolic cages that directed urine through slatted floors into containers containing 20% sulfuric acid (Tobal 1999). A 200 mL subsample was collected twice daily, stored in containers with 20% sulfuric acid, and frozen at −20°C for subsequent nitrogen analysis (method 4.1.2) (VDLUFA 2012).

#### 2.2.3 Statistical Analysis

The statistical analysis was conducted to evaluate the treatment effects on intake of DM, OM, and nitrogen (g/day and g/day per kg metabolic body weight); water intake (L/day); fecal excretion of DM, OM, and nitrogen (g/day and g/day per kg metabolic body weight); urinary nitrogen excretion (g/day and g/day per kg metabolic body weight); nitrogen absorbed (g/day per kg metabolic body weight); nitrogen retention (g/day and g/day per kg metabolic body weight); apparent OM digestibility (%); and the ratios of nitrogen in urine to nitrogen absorbed and nitrogen retained to nitrogen absorbed. For statistical analysis, data were summarized by animals within a period to respect the experimental unit of the crossover design. All analyses were performed using R (version R 4.5.2). Data were analyzed using linear mixed‐effects models, with diet and period included as fixed effects and animal included as a random effect. This structure accounted for the incomplete and unbalanced crossover design. Differences were considered significant at *p* < 0.05, whereas values between 0.05 and 0.10 were considered to indicate a tendency for a treatment effect.

## 3 Results

The CP effective degradability in the fermentative compartment of alpacas showed distinct numerical ranges among feedstuffs (Table 1). Soybean meal presented the highest values, ranging from 91.11% at 2%/h to 68.43% at 8%/h. Alfalfa hay showed high values, with a range from 87.45% to 78.69%. Oat hay presented intermediate values, ranging from 73.19% to 56.52%. Fish meal showed the lowest values, ranging from 47.94% to 37.41%.

The chemical composition and energy concentration of individual feedstuffs are reported in Table 2, whereas the ingredient inclusion and the calculated chemical and nutritional characteristics of the UDCP treatments are presented in Table 3. No differences were observed among treatments in DM intake (g/day), OM intake (g/day), fecal excretion of DM (g/day), OM (g/day), and nitrogen (g/day and g/kg metabolic body weight); nitrogen retention (g/day and g/kg metabolic body weight); and apparent OM digestibility (%) (*p* ≥ 0.33) (Tables 5 and 6). In contrast, water intake (L/day) was influenced by UDCP treatment (*p* = 0.046), with the medium UDCP treatment showing greater water intake than the high UDCP treatment, while the low UDCP treatment did not differ from either group (Table 5). Nitrogen intake (g/day and g/day per kg metabolic body weight) was greater in the low and high UDCP treatments and lowest in the medium UDCP treatment (*p* ≤ 0.004) (Tables 5 and 6). Total urinary nitrogen excretion (g/day and g/kg metabolic body weight) was greater in the low UDCP treatment than in the high UDCP treatment (*p* ≤ 0.01), whereas the medium UDCP treatment did not differ from either of them (Tables 5 and 6).

**TABLE 5:** Dry matter, nitrogen, and organic matter intake, excretion, and digestibility in alpacas subjected to different UDCP treatments.

	Low UDCP		Medium UDCP		High UDCP		UDCP effect *p*
Treatments	Mean	SD	Mean	SD	Mean	SD	
Intake (g/day)							
Dry matter	999.50	161.80	1043.91	187.53	1033.68	184.96	0.53
Nitrogen	16.40^a^	2.66	14.50^b^	2.60	15.69^a^	2.81	0.004
Organic matter	946.98	153.30	992.72	178.33	978.79	175.14	0.50
Water (L/day)	5.35^ab^	0.20	5.46^a^	0.15	5.34^b^	0.19	0.046
Excretion (g/day)							
Fecal dry matter	285.78	50.69	290.53	48.43	273.95	71.99	0.69
Fecal organic matter	258.53	45.59	258.70	43.11	241.37	61.44	0.48
Fecal nitrogen	4.68	1.15	4.81	0.99	4.59	1.47	0.84
Urinary nitrogen	4.92^a^	1.13	4.36^ab^	1.05	4.01^b^	0.79	0.01
Nitrogen retained (g/day)	4.89	2.36	5.94	1.93	5.80	1.70	0.33
Apparent organic matter digestibility (% organic matter)	70.72	6.02	71.58	4.41	73.27	4.75	0.39

*Note:* Values within a row with different superscripts (a, b) indicate differences at *p* < 0.05. A tendency was considered for *p*‐values between 0.05 and 0.10.

Abbreviations: UDCP, undegraded crude protein in the fermentative compartment of the alpaca; SD, standard deviation.

**TABLE 6:** Nitrogen intake, excretion, absorption, and retention (g/day per kg metabolic body weight), and nitrogen efficiency ratios in alpacas subjected to different UDCP treatments.

	Low UDCP		Medium UDCP		High UDCP		*Diet effect p*
	Mean	SD	Mean	SD	Mean	SD	
Nitrogen intake	0.84^a^	0.11	0.74^b^	0.10	0.80^a^	0.12	0.003
Fecal nitrogen	0.24	0.06	0.25	0.05	0.24	0.07	0.81
Nitrogen absorbed	0.60^a^	0.14	0.49^b^	0.10	0.57^ab^	0.07	0.01
Urinary nitrogen	0.25^a^	0.06	0.22^ab^	0.05	0.21^b^	0.04	0.009
Nitrogen retained	0.25	0.12	0.30	0.09	0.30	0.09	0.33
Ratio of nitrogen in urine: nitrogen absorbed	0.44^ab^	0.14	0.47^a^	0.12	0.37^b^	0.09	0.03
Ratio nitrogen retained: nitrogen absorbed	0.41^b^	0.13	0.62^a^	0.14	0.52^a^	0.12	0.001

*Note:* Values within a row with different superscripts (a, b) differ significantly (*p* < 0.05). A tendency was considered for *p*‐values between 0.05 and 0.10.

Abbreviations: UDCP, undegraded crude protein in the fermentative compartment of the alpaca; SD, standard deviation.

Nitrogen absorbed (g/day per kg metabolic body weight) was greater in the low UDCP treatment compared with the medium UDCP treatment, whereas the high UDCP treatment did not differ from either the low or the medium UDCP treatment (*p* = 0.01) (Table 6). The ratio of urinary nitrogen to absorbed nitrogen was influenced by UDCP treatment (*p* = 0.03), with the medium UDCP treatment showing a greater ratio than the high UDCP treatment, while the low UDCP treatment did not differ from either of them. The ratio of retained nitrogen to absorbed nitrogen was also influenced by UDCP treatment (*p* = 0.001), with both the medium and high UDCP treatments showing greater ratios than the low UDCP treatment (Table 6).

## 4 Discussion

The present study was conducted under controlled experimental conditions using an incomplete and unbalanced crossover design due to logistical constraints related to the limited availability of metabolic cages. This structure reduced the number of observations in some treatment–period combinations and increased the risk of confounding among treatment, period, and animal effects. To minimize these limitations, data were summarized by animal within period to respect the experimental unit, and linear mixed‐effects models were applied, including diet and period as fixed effects and animal as a random effect. This approach accounted for repeated measurements within animals, adjusted for temporal variation among periods, and accommodated the unequal number of observations per treatment, thereby reducing bias associated with the incomplete and unbalanced allocation of treatments.

In addition, the experimental diets included feed ingredients that are not commonly used in practical alpaca feeding systems. This approach was intentional and reflects the experimental nature of the study, which aimed to generate diets with clearly differentiated UDCP proportions to isolate its effects on nitrogen partitioning. Consequently, the results should be interpreted primarily in a physiological and mechanistic context, serving as a basis for evaluating whether adjustments in CP degradability merit consideration in future nutritional recommendations for alpacas. With these constraints in mind, the following sections interpret the responses in a physiological and mechanistic context.

### 4.1 Voluntary Intake, Fecal Excretion, and Apparent Digestibility

In the present study, DM intake ranged from 798 to 1316 g/day, corresponding to 1.56%–2.37% of body weight, which falls within the range of 1.4%–2.8% of body weight previously reported for alpacas (NRC 2007; Gómez‐Oquendo et al. 2024). No differences in DM intake were observed among UDCP treatments (*p* = 0.53). This response was expected, as the experimental diets were isoenergetic and voluntary DM intake in alpacas is more strongly regulated by dietary energy concentration than by CP degradability (San Martín and Van Saun 2014). A similar response was reported by Lund et al. (2012), who observed no effect of different UDCP proportions on DM intake in alpacas.

The lower nitrogen intake observed in the medium UDCP treatment (*p* = 0.004) occurred despite similar dietary CP concentrations among treatments and can be explained by differences in ingredient composition. The low and high UDCP treatments contained higher proportions of soybean meal and fish meal, respectively, whereas these nitrogen‐rich ingredients were included at intermediate levels in the medium UDCP treatment. South American camelids selectively consume diet components with higher nitrogen concentration when heterogeneous feeds are offered (San Martín and Bryant 1989; Van Saun 2006). Consequently, the greater availability of nitrogen‐rich ingredients in the low and high UDCP treatments allowed a higher absolute intake of these components, resulting in greater nitrogen intake, whereas selection in the medium UDCP treatment was constrained by the lower proportion of concentrate offered. No differences were observed among treatments in OM intake (*p* = 0.50), which was expected given the similar DM intake and the comparable OM concentration of the experimental treatments (972–975 g/kg DM).

Water intake differed among UDCP treatments, with the medium UDCP diet showing higher values than the high UDCP diet, whereas the low UDCP diet did not differ from either treatment. This difference was not explained by DM intake or by absolute urinary nitrogen excretion, which were comparable among treatments (Tables 5 and 6). Instead, the medium UDCP treatment exhibited the highest ratio of urinary nitrogen to absorbed nitrogen, indicating lower efficiency of post‐absorptive nitrogen utilization. In South American camelids, water intake is closely associated with renal solute excretion and osmotic regulation (Van Saun 2014; Gerken et al. 2019). Consequently, a greater proportion of absorbed nitrogen excreted via urine would increase the relative renal solute load, thereby increasing water requirements for urinary nitrogen elimination.

Fecal DM and OM excretion did not differ among treatments (*p* ≥ 0.48), nor did apparent OM digestibility (*p* = 0.39). This response was expected, as DM intake (*p* = 0.53) and OM concentration of the diets (coefficient of variation = 0.18% of the mean) were similar across treatments, resulting in comparable OM intake (*p* = 0.50) and fecal OM output (*p* = 0.48). Under isoenergetic conditions (coefficient of variation = 1.09% of the mean), with similar fiber concentrations (coefficient of variation = 0.96% of the mean) and DM intake, variations in UDCP proportion affected nitrogen metabolism without altering apparent OM digestibility.

### 4.2 Nitrogen Excretion, Retention, and Utilization

Fecal nitrogen excretion did not differ among treatments (*p* = 0.84). Fecal nitrogen originates from undigested dietary nitrogen, endogenous losses, and microbial nitrogen (Mason and Frederiksen 1979). Endogenous losses are relatively constant (GfE 2001), and microbial CP production is primarily driven by dietary energy supply (GfE 2001), which was similar among treatments. Although UDCP proportion altered the site of nitrogen release along the digestive tract, total CP digestion was not affected, resulting in comparable fecal nitrogen excretion across treatments.

In contrast, urinary nitrogen excretion differed between treatments (*p* ≤ 0.01), decreasing as dietary UDCP increased. This pattern indicates an improvement in nitrogen utilization with increasing UDCP proportion, as a smaller fraction of absorbed nitrogen was excreted via urine. Consistent with this response, alpacas receiving diets with 0% UDCP have been reported to spend more time urinating, reflecting greater urinary nitrogen losses and reduced nitrogen recycling within the fermentative compartment (Lund et al. 2012).

Nitrogen retention expressed as an absolute amount did not differ among treatments (*p* = 0.33), indicating that total retained nitrogen alone does not adequately describe nitrogen use efficiency. Differences among treatments became evident when nitrogen retention and urinary nitrogen losses were evaluated relative to absorbed nitrogen (*p* ≤ 0.03). Although nitrogen absorption was greater in the low UDCP treatment due to higher intake and similar fecal nitrogen excretion, a larger proportion of absorbed nitrogen was lost via urine in both the low and medium UDCP treatments. In contrast, the high UDCP treatment exhibited the lowest urinary nitrogen loss relative to absorbed nitrogen, indicating a more efficient post‐absorptive utilization of nitrogen. When nitrogen retention was expressed relative to absorbed nitrogen, both the medium and high UDCP treatments showed higher retention ratios than the low UDCP treatment. However, the higher urinary nitrogen loss relative to absorption observed in the medium UDCP treatment indicates reduced efficiency compared with the high UDCP treatment. Therefore, considering both urinary nitrogen losses and retention efficiency, the high UDCP treatment can be identified as the most efficient in terms of nitrogen utilization.

An additional perspective on nitrogen utilization among treatments can be obtained by considering the balance between dietary energy and protein supply. When estimated using the old German ruminant system (GfE 2001), the rumen nitrogen balance (RNB) of the experimental diets was 2.82, 0.28, and −1.40 g/kg DM for the low, medium, and high UDCP treatments, respectively. A slightly negative RNB, as observed for the high UDCP treatment, is generally interpreted in ruminants as indicative of a more efficient nitrogen utilization, as it reflects a closer synchronization between fermentable energy and rumen‐available nitrogen.

However, this interpretation is subject to several important limitations. First, the RNB concept and calculation were developed for ruminants and are based on CP supply relative to utilizable CP, defined as the sum of UDCP and microbial CP (GfE 2001). In the present study, microbial CP was estimated using the standard relationship of 10.1 g microbial CP per MJ of metabolizable energy, a value derived from ruminant systems. In addition, metabolizable energy was not directly measured but estimated from the literature, which further constrains the precision of absolute RNB values. Despite these limitations, the experimental diets were isoenergetic, and any adjustment of microbial CP yield coefficients specific to alpacas would be expected to affect absolute RNB values rather than the relative differences among treatments. Therefore, the more negative RNB estimated for the high UDCP treatment is consistent with the observed reduction in urinary nitrogen losses and supports the conclusion of improved nitrogen utilization efficiency at higher UDCP concentrations.

The present study demonstrates that modifying the proportion of UDCP in the fermentative compartment influences nitrogen utilization efficiency in alpacas without affecting voluntary intake, fecal excretion, or apparent OM digestibility when diets are isoproteic and isoenergetic. Increasing UDCP proportion (~34%) reduced urinary nitrogen losses and improved the efficiency of post‐absorptive nitrogen utilization. From a practical and physiological perspective, these findings indicate that CP degradability is a relevant nutritional factor in alpacas and should be considered alongside CP concentration when formulating diets. The results support the need to develop UDCP‐based nutritional recommendations for alpacas to improve nitrogen efficiency and reduce nitrogen losses. Further research is recommended to evaluate the interaction between UDCP proportion and fermentable energy supply, as well as the long‐term effects of balanced CP fractions on productivity, reproduction, and environmental nitrogen losses in alpaca production systems.

## Funding

This work was supported by Universidad Científica del Sur, 10.13039/100020469, No. 003‐DGIDI‐2023.

## Conflicts of Interest

The authors declare no conflicts of interest.

## Data Availability

The data that support the findings of this study are available from the corresponding author upon reasonable request.
